# Branch Retinal Artery Occlusion Following Rhinoplasty: A Case Report

**DOI:** 10.7759/cureus.42265

**Published:** 2023-07-21

**Authors:** Saleh S Algamdi, Askar K Alshaibani, Wajeeha I Alkhars, Khalid Alghamdi

**Affiliations:** 1 Department of Ophthalmology, College of Medicine, Imam Abdulrahman Bin Faisal University, Dammam, SAU; 2 Department of Ophthalmology, Dhahran Eye Specialist Hospital, Dhahran, SAU; 3 College of Medicine, King Saud Bin Abdulaziz University for Health Sciences, Jeddah, SAU

**Keywords:** case report, complications, rhinoplasty, septoplasy, branched retinal artery occlusion

## Abstract

Central retinal artery occlusion (CRAO) is a sudden and vision-threatening condition with catastrophic consequences unless managed immediately by reestablishing the retinal circulation. Even though CRAO is a common ocular disorder, it is a very rare complication after non-ocular surgeries; only a few cases have been reported in the literature. Cardiac and spine surgeries are considered the most common causes of postoperative vision loss.

In this case report, we present the case of a young female patient diagnosed with central retinal artery occlusion after a septoplasty. This is considered the first reported case in the Kingdom of Saudi Arabia. Branch retinal artery occlusion (BRAO) and CRAO are possible complications of otorhinolaryngology procedures, and both otorhinolaryngologists and ophthalmologists should be aware of these possible complications.

## Introduction

Retinal artery occlusion is a sudden, abrupt, and usually, irreversible disorder where a patient can end up with visual loss due to ischemic damage unless a re-establishment of the retinal circulation is immediately performed. Central retinal artery occlusion (CRAO) was first described by Von Graefes in 1859 [1]. Even though retinal artery occlusion is a common ocular-vascular disorder with an incidence of 8.5 per 100,000, the incidence of the condition after non-ocular operative interventions is greater (0.013%) [2, 3]. The most common non-ocular procedures that may lead to vision loss are cardiac and spine surgeries, possibly due to a wide variety of mechanisms, including anterior or posterior ischemic optic neuropathy, retinal artery occlusion, pituitary apoplexy, or even cortical blindness [3].

Visual defects after rhinoplasty usually happen after corticosteroids (or other medications) are injected into the nasal cavity. Additionally, local anesthetics containing epinephrine have been linked to this visual defect [4]. Central retinal artery occlusion can lead to catastrophic vision loss, with 80% of patients ending up with 20/400 visual acuity or worse [5]. In this article, we share our experience of a rare case of a young female patient who presented with the chief complaint of a sudden, painless decrease in vision for a week following rhinoplasty four days prior to the onset of symptoms.

## Case presentation

A 29-year-old female patient with no significant medical history presented to the emergency department at the Dhahran Eye Specialist Hospital in Dhahran, Saudi Arabia, complaining of a sudden, painless decrease in vision in the left eye for the last week. She did not have any ocular surgeries or diseases in the past, and her only past surgical history was a rhinoplasty performed four days prior to the onset of her symptoms. The surgery was uncomplicated, but the patient reported blurry vision for an hour following the procedure, which resolved spontaneously. The patient did not seek any kind of medical assistance when the symptoms started.

**Figure 1:**
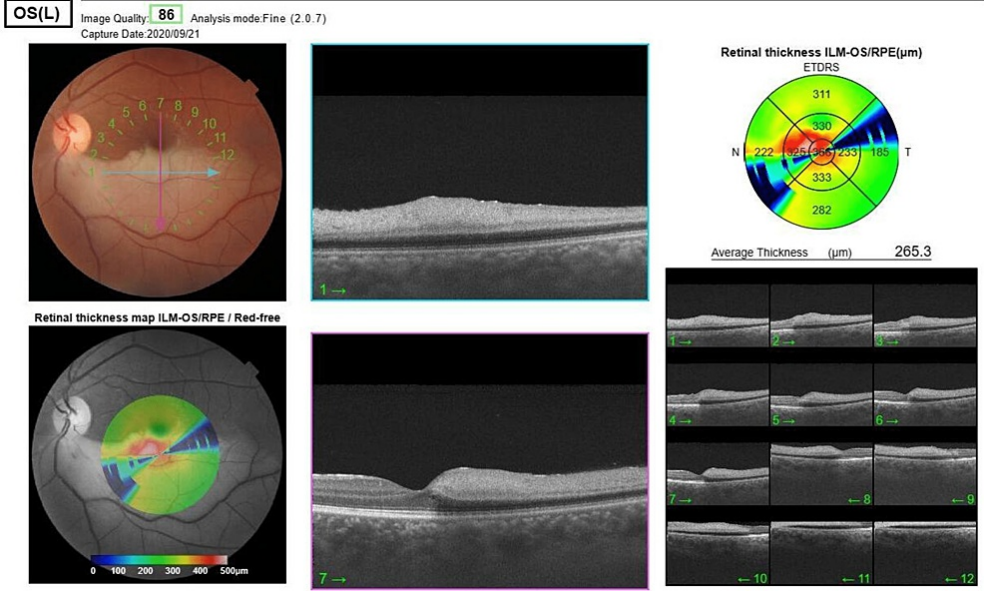
Optical coherence tomography of the left eye showed thickening of the inner part of the inferior hemiretina with a shadowing effect, indicating edema.

On examination, visual acuity (VA) without correction was 20/22 in the right eye and 20/400 in the left eye; the intraocular pressure (IOP) was 12 mmHg and 13 mmHg in the right eye and left eye, respectively. The anterior chamber was deep and quiet in both eyes. The pupil was round, regular, and reactive to light in the right eye; however, the left eye displayed a relative afferent pupillary defect (RAPD). Furthermore, there was no neovascularization of the iris in both eyes, and the lenses were clear in both eyes. On fundus examination, the right eye showed a normal retina with a dry macula and a healthy disk; in the left eye, there was whitening of the inferior hemiretina involving the macula along the intertemporal artery, indicating edema. Optical coherence tomography (OCT) was performed and showed thickening of the inner part of the inferior hemiretina with a shadowing effect, indicating edema (Figure 1).

**Figure 2:**
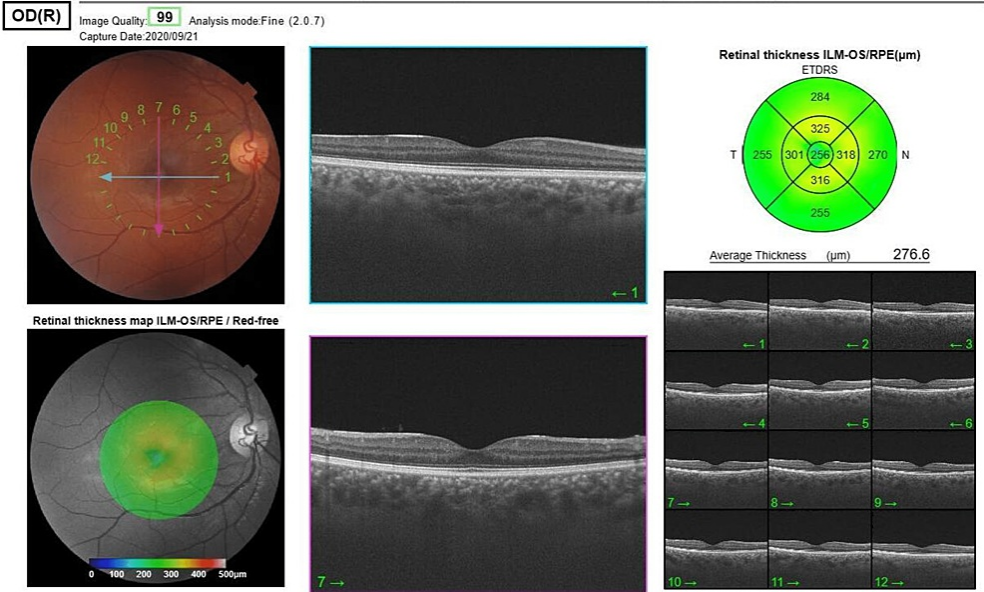
Normal optical coherence tomography of the right eye.

Figure 2 shows the normal macular OCT of the other eye.

**Figure 3:**
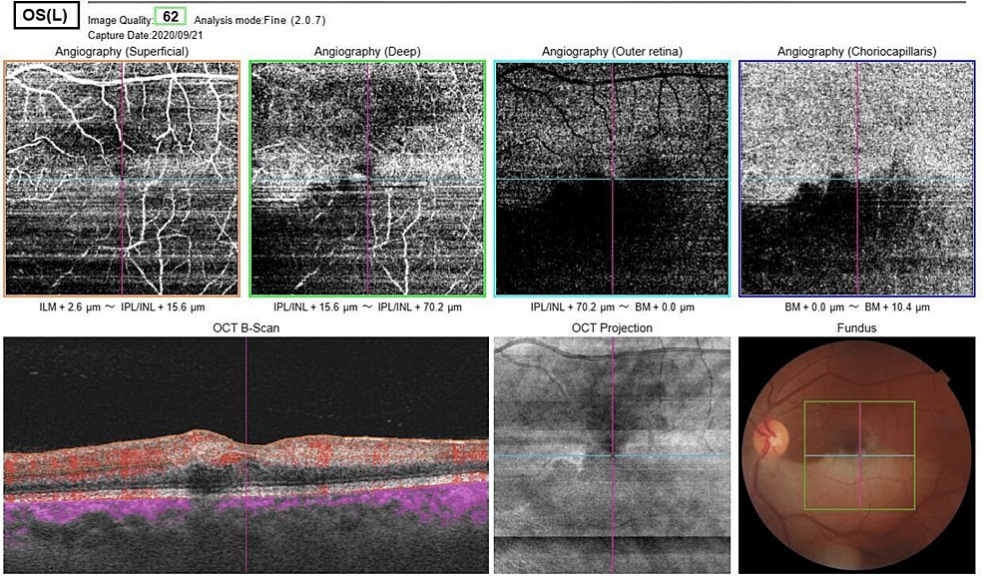
Optical coherence tomography angiography of the left eye detected no capillary blood flow in the affected area.

**Figure 4:**
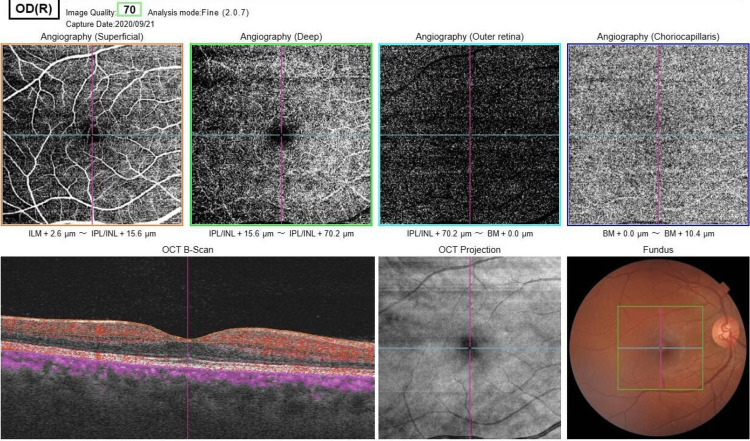
Normal optical coherence tomography angiography of the right eye.

Additionally, OCT-angiography revealed a lack of capillary blood flow in the affected area (Figure 3) and a normal study in the other eye (Figure 4).

Based on the above-mentioned findings, the main suspicion was branch retinal artery occlusion (BRAO) in the left eye. Therefore, fundus fluorescein angiography (FFA) and a visual field study were requested, and the patient was referred to the cardiology department to rule out thromboembolic disorders. The FFA showed delayed filling in the inferior arcade of the left eye, which typically begins roughly 3.5 minutes even with a minimal flow. A visual field (VF) study revealed a superior hemifield defect in the left eye and normal VF in the right eye.

Echocardiography was performed by a cardiologist and showed a normal echo-Doppler study. The patient was ultimately diagnosed with BRAO, possibly as a complication of rhinoplasty, and a follow-up appointment was scheduled. No management or treatment was delivered to the patient due to the late presentation. After one month, the patient returned for her follow-up appointment. Her VA had dramatically improved to 20/50 in the left eye after the resolution of the macular edema, but she had a persistent superior visual hemifield defect.

## Discussion

Retinal artery occlusion is a sudden, abrupt cessation of the retinal circulation, either to all or some parts of the retina, which can lead to hypoperfusion and ischemic damage. Retinal artery occlusion has a wide variety of causes related to non-ocular operations, most commonly cardiac and spine surgeries [3]. In our case, the patient didn’t report any known cause that may have led to this condition except for the rhinoplasty, which was performed a few days prior to the onset of her symptoms.

In the literature, there are a few similar reported cases of CRAO and BRAO after rhinoplasty surgeries. Nageswar et al. reported a similar case of a patient who developed CRAO and third cranial nerve palsy following nasal septoplasty. In contrast to our patient, their case reported BRAO in addition to third cranial nerve involvement [6]. A case reported by Leng et al. involved a patient who presented with an inferior visual field defect in his right eye immediately after septoplasty. In contrast to our patient, who developed symptoms a few days after the surgery. The researcher mentioned that a similar case could be caused by either direct trauma or spastic or embolic vascular events [4]. Chowdhary et al. reported another case of a 25-year-old patient who presented to the hospital complaining of sudden, painless visual loss in the left eye. They managed the case using hyperbaric oxygen therapy four days after the insult. Despite the long period after the insult, which may be considered out of the window for intervention, there was an improvement both in the visual acuity and the visual field [7].

Central retinal artery occlusion has been reported as a rare complication after some otorhinolaryngology procedures. While this complication is not fully understood, it has been linked to different mechanisms, including trauma to the orbital wall or structures, injection of local anesthetic during the procedure, and fat embolism, which is considered an extremely rare complication [5, 7, 8]. Vasoconstrictive agents such as adrenaline are commonly used as local anesthetics during otorhinolaryngology procedures. They are used to help decrease bleeding during the procedure and improve the view of the surgery area. Although they are a commonly used and helpful medication intraoperatively, vasoconstrictive agents have many side effects related to the cardiovascular system. It is proposed that the use of adrenaline may cause vasospasms and obstruction of the ophthalmic and retinal arteries.

This complication, which may lead to vision loss, can be avoided by adding topical vasoconstrictive medications to the mucosa before injecting the anesthetic medication. Another way to avoid this complication is by using slow injection and aspiration before injecting the anesthetic agent to avoid an unwanted intraarterial injection [4].

## Conclusions

In this case report, we discussed one of the rare complications of otorhinolaryngology surgeries, which both otorhinolaryngologists and ophthalmologists should know. Early diagnosis and management of such complications may prevent serious sequelae for the patient that could have long-term effects on the patient and his family. This is the first reported case of branch retinal artery occlusion after rhinoplasty in the Kingdom of Saudi Arabia.
